# Unveiling Potential Mechanisms of Spatholobi Caulis against Lung Metastasis of Malignant Tumor by Network Pharmacology and Molecular Docking

**DOI:** 10.1155/2022/1620539

**Published:** 2022-03-21

**Authors:** Feiyu Xie, Mina Wang, Yixin Su, Kunmin Xiao, Xuelei Chu, Sidan Long, Linlu Li, Xin Zhang, Peng Xue, Shijie Zhu

**Affiliations:** ^1^School of Traditional Chinese Medicine, Beijing University of Chinese Medicine, Beijing 100029, China; ^2^Oncology Department, Wangjing Hospital of China Academy of Chinese Medical Sciences, Beijing 100102, China; ^3^Department of Acupuncture and Moxibustion, Beijing Hospital of Traditional Chinese Medicine, Beijing Key Laboratory of Acupuncture Neuromodulation, Capital Medical University, Beijing 100010, China; ^4^School of Biological Sciences, Nanyang Technological University, 60 Nanyang Drive, Singapore 637551

## Abstract

**Background:**

Lung metastasis of malignant tumor signifies worse prognosis and immensely deteriorates patients' life quality. Spatholobi Caulis (SC) has been reported to reduce lung metastasis, but the mechanism remains elusive.

**Methods:**

The active components and corresponding targets of SC were obtained from the Traditional Chinese Medicine Database and Analysis Platform (TCMSP) database and the SwissTargetPrediction database. The disease targets were acquired from DisGeNET and GeneCards databases. Venn map was composed to figure out intersection targets by using R. The PPI network was constructed through STRING and Cytoscape, and MCODE plug-in was used to sift hub targets. Gene Ontology (GO)-Kyoto Encyclopedia of Genes and Genomes (KEGG) enrichment analysis was carried out by utilizing clusterProfiler package (R3.6.1) with adjusted *P* value <0.05. Network of SC-active components-intersection targets-KEGG pathway was accomplished with Cytoscape. Molecular docking between hub targets and active components was performed, analyzed, and visualized by AutoDockTools, AutoDock Vina, PLIP Web tool, and PYMOL.

**Results:**

24 active components and 123 corresponding targets were screened, and the number of disease targets and intersection targets was 1074 and 47, respectively. RELA, JUN, MAPK1, MAPK14, STAT3, IL-4, ESR1, and TP53 were the 8 hub targets. GO analysis and KEGG analysis elucidated that SC could ameliorate lung metastasis mainly by intervening oxidative stress, AGE-RAGE signaling pathway, and microRNAs in cancer. All 8 hub targets were proven to combine successfully with active components of SC.

**Conclusion:**

Inflammation is the core factor that integrates all these targets, biological process, and signaling pathways, which indicates that SC prevents or reduces lung metastasis mainly by dispelling inflammation.

## 1. Introduction

It is estimated that the number of worldwide new cancer cases and cancer deaths has exceeded 19 million and 10 million, respectively, in 2020 [1], which caused an immense burden on individuals and medical systems. More than 90% of death cases are closely associated with metastasis of malignant tumor, and the lung is one of the most common target organs for distant metastasis in various cancer types [2–5]. Since the aroused complications, like pleural effusion, superior vena cava syndrome, and failure of respiratory and circulatory systems tremendously impair patients' quality of daily life, it is of great urgency and necessity to explore effective measures to prevent or reduce lung metastasis [6].

The question of why metastatic cancer cells manifest the feature of organotropism has brought about extensive discussions. Paget put forward the “seed and soil” hypothesis that prometastatic tumor cells (the “seed”) colonize in specific organ sites (the “soil”) where the microenvironment is favorable for metastasis [7, 8]. In 2005, Kaplan et al. [9] proposed the concept of a premetastatic niche that renders distant organs hospitable for disseminating tumor cells to colonize through intricate mechanisms involving different cells, chemokines, proteins, cytokines, exosomes, etc. Traditional Chinese medicine is specialized in intervening multiple targets and pathways simultaneously, which might be a promising therapeutic strategy for lung metastasis.

Spatholobi Caulis (SC), the vine stem of *Spatholobus suberectus* Dunn., is a typical kind of traditional Chinese medicine that possesses functions of invigorating blood circulation and eliminating stasis. It has been proven that 80% ethanol extracts of SC significantly reduced the pulmonary metastasis and prolonged the life span of Balb/c mice, which had been injected with 4T1-luc cells [10]. The underlying mechanism might be related to the decrease of tumor-platelet complex formation, indicating that SC may inhibit lung metastasis by alleviating the hypercoagulation state. However, SC is composed of complicated ingredients and its anti-lung metastasis mechanism may be far beyond imagination, which needs further exploration. Thus, we integrated the technology of network pharmacology and molecular docking to better discover the inherent mechanisms.

## 2. Materials and Methods

### 2.1. Screening the Active Components of SC and Corresponding Targets

With the thresholds of oral bioavailability (OB) ≥30% and drug-likeness (DL) ≥0.18, the active components of SC and its corresponding targets were obtained from the Traditional Chinese Medicine Database and Analysis Platform (TCMSP) (https://tcmsp-e.com/) [11]. To those active components without targets in the TCMSP, the SwissTargetPrediction database (http://new.swisstargetprediction.ch/) [12] was utilized to predict their potential targets after acquiring 2D structures in “SDF” files from the PubChem database (https://pubchem.ncbi.nlm.nih.gov/) [13]. To ensure the accuracy of the predicted targets, the following three requirements had to be satisfied: (i) only top 15 targets would be chosen; (ii) the probability score should not be less than 0.7; (iii) relative research literature were retrieved to verify whether the active component could regulate predicted targets effectively, which would be otherwise deleted. Finally, the targets of SC were converted to standardized gene symbols in the UniProt database (http://www.uniprot.org/uniprot/) [14].

### 2.2. Genes and Proteins Related to Lung Metastasis of Malignant Tumor

The academic name of lung metastasis “secondary malignant neoplasm of lung” was searched in the DisGeNET database (http://www.disgenet.org) [15] and GeneCards database (https://www.genecards.org/) [16]. With the species limited to “*Homo sapiens*,” only those targets that appear in both databases simultaneously were chosen as targets relevant to lung metastasis of malignant tumor.

### 2.3. Intersection Targets

Before using *R* (v3.6.1) software (Statistics Department of the University of Auckland, New Zealand), we created two files in “csv” format to store the targets of the active components of SC and targets relevant to lung metastasis of malignant tumor, respectively. The two files were read by *R* (v3.6.1) software, and the VennDiagram package was employed to get the intersection targets between SC and lung metastasis.

### 2.4. Protein-Protein Interaction (PPI) Analysis of Intersection Targets and Hub Targets

Setting minimum required interaction score as 0.9, the intersection targets were imported into STRING 11.0 [17] to gain the PPI data, which were subsequently processed through Cytoscape 3.7.2, and hub targets were picked by means of MCODE plug-in of Cytoscape after fixing the degree cutoff to 2 and node score cutoff to 0.2 [18, 19].

### 2.5. GO Enrichment Analysis and KEGG Pathway Analysis of Intersection Targets

Gene Ontology (GO) functional enrichment analysis and Kyoto Encyclopedia of Genes and Genomes (KEGG) pathway analysis of intersection targets were conducted to show the top 15 items by using clusterProfiler package (R3.6.1) with adjusted *P* value <0.05 [20].

### 2.6. Network Construction of SC-Active Components-Intersection Targets-KEGG Pathway

This network was constructed by Cytoscape 3.7.2 to visualize mechanisms of SC treating lung metastasis.

### 2.7. Molecular Docking between Hub Targets and Active Components

We followed three key principles to select the most suitable 3D structures of the hub targets: (i) the source organism should be “*Homo sapiens*”; (ii) the original 3D structures should possess at least one eutectic ligands; and (iii) only the 3D structures with the least “resolution” value would be chosen. The selected 3D structures of the hub targets were downloaded from the PDB database (http://www.rcsb.org/) [21], and removal of water molecules and ligands of the targets was carried out with the help of PYMOL (DeLano Scientific Limited Liability Company, South San Francisco, USA). Meanwhile, the 3D structures of the active components were collected from the PubChem database (https://pubchem.ncbi.nlm.nih.gov/) [22] and were transferred into MOL2 format by using OPENBABEL software (Free Software Foundation, Inc. 51 Franklin St, Fifth Floor, Boston, MA 02110–1301 USA). AutoDockTools (v1.5.6; Department of Molecular Biology, The Scripps Research Institute, La Jolla, California, USA) was used to complete preparation work for docking, including combining nonpolar hydrogen and distributing charge for active components, adding polar hydrogens, and distributing charges for hub targets. It should be noted that we established a docking box for each hub target, which was big enough to cover the whole macromolecular protein to make sure that every possible functional pocket would be simulated to accommodate each active component for molecular docking. Eventually, the docking was performed via AutoDock Vina (v1.1.2) software (Department of Molecular Biology, The Scripps Research Institute, La Jolla, California, USA). Based on the affinity and the number of hydrogen bonds formed, we chose the most practical conformation of each hub target and output complexes of hub targets and active components in “pdb” format with the aid of PYMOL. Finally, all the complexes were uploaded to PLIP Web tool (https://plip-tool.biotec.tu-dresden.de/plip-web/plip/index) [23] to analyze and visualize other noncovalent interactions between hub targets and active components.

## 3. Results

### 3.1. Active Components of SC and Corresponding Targets

According to the criterion, 24 active components were recorded, and 123 targets of the active components were identified. The basic information of 24 active components is listed in Table 1.

### 3.2. Targets of Lung Metastasis of Malignant Tumor and Intersection Targets

**Figure 1:**
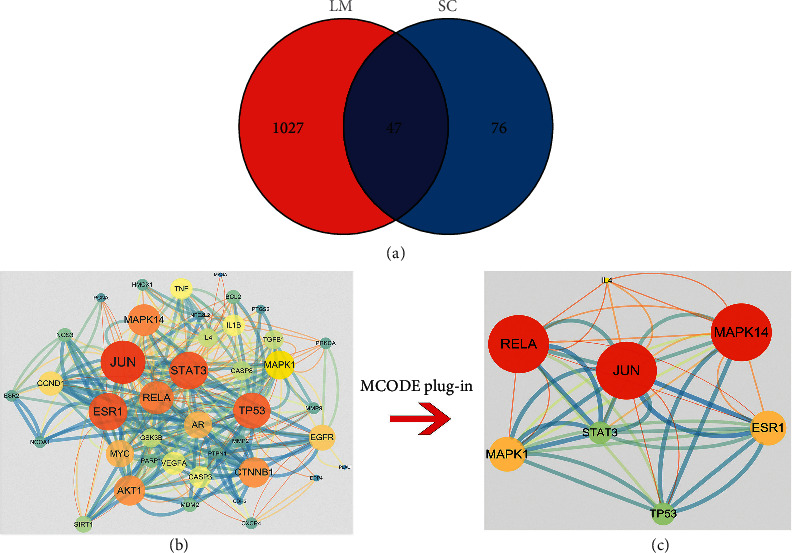
(a) Intersection targets of SC and secondary malignant neoplasm of lung. (b) PPI network of intersection targets. (c) PPI network of hub targets.

There were altogether 1074 targets that both existed in the 2 databases, among which 47 targets were intersection targets after accomplishing Venn map by using *R* software (Figure 1(a)).

### 3.3. PPI Network of Intersection Targets and Sifting of Hub Targets

After hiding disconnected nodes in the STRING database, the original PPI network exhibited 41 nodes and 165 edges and was imported to Cytoscape ((v 3.7.2) for further process, shown in Figure 1(b). Afterwards, we applied MCODE plug-in to sift hub targets, which are displayed in Figure 1(c). Degree represents the number of edges of the certain target and the higher the degree is, the larger and the brighter the target shows, implying the significance of the target. The comprehensive score was used to evaluate the relationship between targets. The higher score corresponds to the thicker and darker edge in the image, indicating that the relationship between targets is closer.

### 3.4. GO Enrichment Analysis and KEGG Pathway Analysis of Intersection Targets

**Figure 2:**
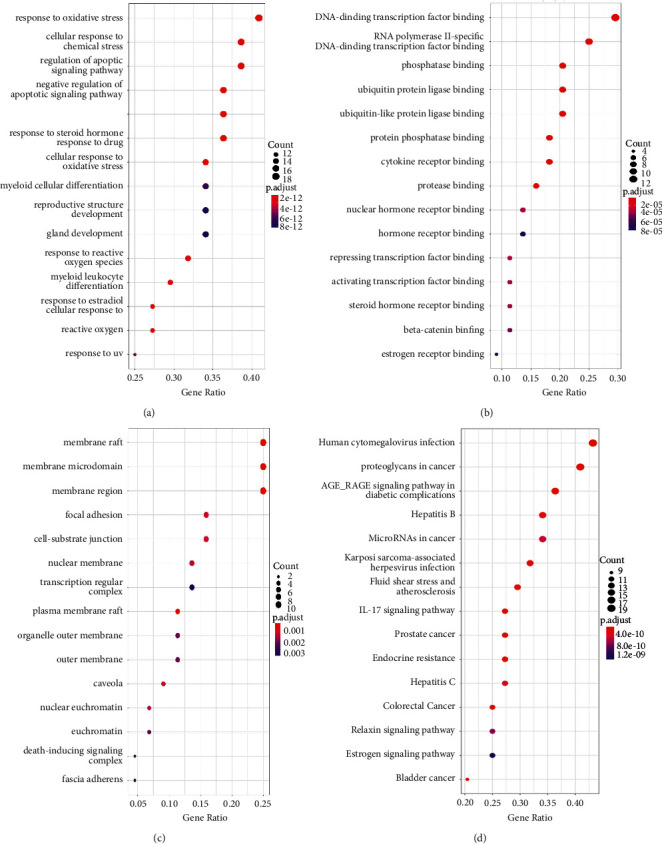
GO enrichment analysis and KEGG pathway analysis of the intersection targets: (a) biological process; (b) molecular function; (c) cellular component; (d) Kyoto Encyclopedia of Genes and Genomes pathway analysis.

With *p*. adjust ＜0.05, 1846 items of biological process (BP) were acquired, and the top 15 items are illustrated in Figure 2(a), which were closely related to oxidative stress, chemical stress, and apoptosis. Similarly, a total of 83 items of molecular function (MF) and 65 items of cellular component (CC) were obtained, and the top 15 items are shown in Figures 2(b)–2(c), which were tightly related to DNA/RNA activity and components of membrane, respectively. Items of KEGG pathway enrichment analysis amounted to 155, and the AGE-RAGE signaling pathway and microRNAs in cancer were the most relevant to lung metastasis among the top 15 items, which are displayed in Figure 2(d).

### 3.5. Network Construction of SC-Active Components-Intersection Targets-KEGG Pathway

**Figure 3:**
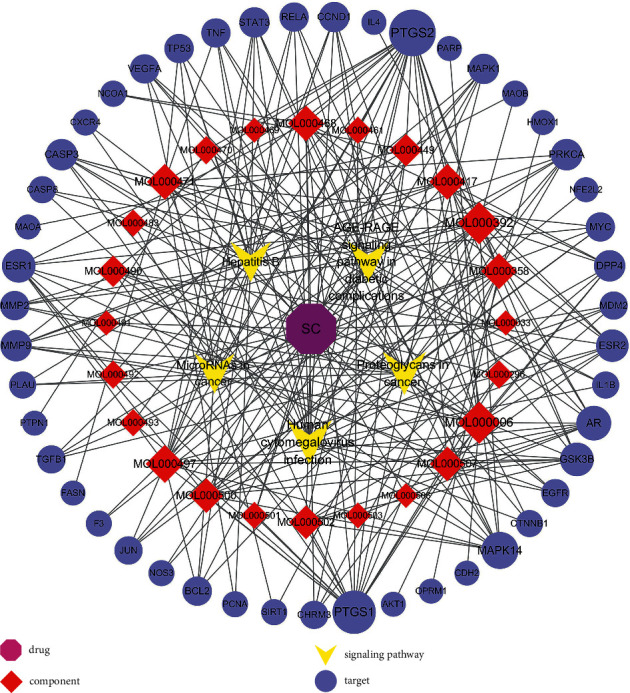
Network of SC-active components-intersection targets-KEGG pathway.

Taking the advantage of Cytoscape, we created the network of SC, 24 active components, 47 intersection targets, and top 5 signaling pathways, containing 77 nodes and 248 edges (shown in Figure 3). The size of items represents degree of value, which means the bigger the item, the higher its degree.

### 3.6. Molecular Docking between Hub Targets and Active Components

**Figure 4:**
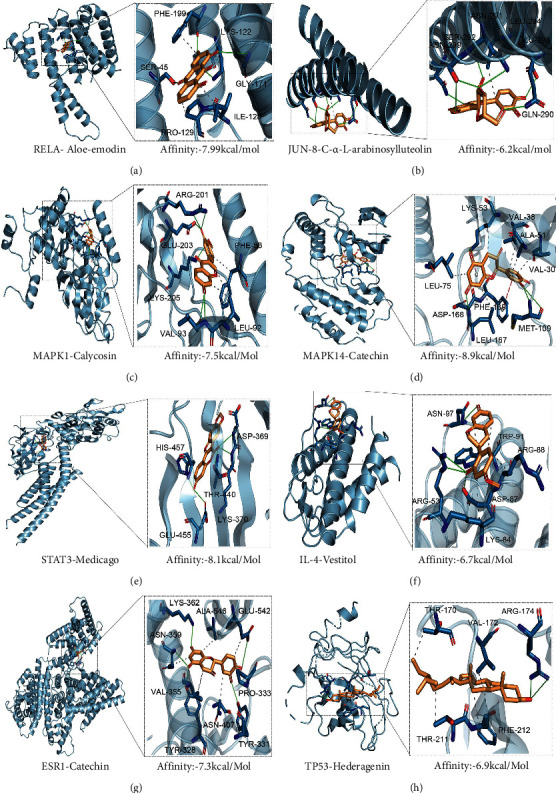
Molecular docking of hub targets and active components: (a) RELA-Aloe-emodin; (b) JUN-8-C-*α*-L-arabinosylluteolin; (c) MAPK1-Calycosin; (d) MAPK14-Catechin; (e) STAT3-Medicago; (f) IL-4-Vestitol; (g) ESR1-Catechin; and (h) TP53-Hederagenin.

In consideration of affinity and hydrogen bonds, we ultimately chose the most practical conformation for each hub target, which were RELA-Aloe-emodin, JUN-8-C-*α*-L-arabinosylluteolin, MAPK1-Calycosin, MAPK14-Catechin, STAT3-Medicago, IL-4-Vestitol, ESR1-Catechin, and TP53-Hederagenin. After analyzing noncovalent interactions, details including affinity, hydrogen bonds, hydrophobic interactions, *π*-stacking, *π*-cation interactions, and relative amino acid residues are exhibited in Supplementary Table 1 and Figure 4. To better illustrate details of molecular docking, hub targets and active ingredients were marked with blue and yellow, respectively, with stick structure of relative residues shown in blue as well. Hydrogen bonds and hydrophobic interactions were displayed by green solid line and grey dotted line separately, meanwhile, *π*-stacking and *π*-cation interactions were present by red and purple dotted line, respectively.

Affinity is the concept that measure binding force between ligand and macromolecular protein, and it is widely acknowledged that the spontaneous combination is likely to occur when the affinity is smaller than −5 kcal/mol. In our research, affinity of all 8 conformations was smaller than −5 kcal/mol and MAPK14-Catechin exhibited the strongest binding force with affinity reaching −8.9 kcal/mol. The formation of hydrogen bonds mainly results from electrostatic force, which stems from charge transfer between donor and acceptor. In our research, at least 2 hydrogen bonds took shape, which offered powerful electrostatic force to form stable conformation, and JUN-8-C-*α*-L-arabinosylluteolin was supported by 7 hydrogen bonds, which is the most among all 8 conformations. Hydrophobic interaction is the force that reduces the free energy and stabilizes the contact between two nonpolar regions. Except for STAT3-Medicago, at least 1 hydrophobic interaction formed in the remaining 7 conformations, and there were 7 hydrophobic interactions formed between ESR1 and catechin, ranking 1^st^ in our research. MAPK14-Catechin was the only conformation that contains *π*-stacking and *π*-cation interactions, which made a great contribution for the stable combination.

## 4. Discussion

Compared with primary tumor, the colonies of lung metastasis often mean worse prognosis and less effectiveness of therapeutic plans. Therefore, preventing or reducing lung metastasis of malignant tumor will extend life expectancy and mitigate the distress of patients with advanced stages of tumor.

In this study, 24 active components were screened in the aggregate, among which many components have been confirmed to suppress lung metastasis with convincing proof. *β*-Sitosterol is a bioactive phytosterol compound that is naturally present in the plant cell membrane, and it was proven to decrease lung metastasis in PC-3 and 4T1 cells *in vivo* and *in vitro* experiments [24–26]. Imanaka et al. [27] discovered that after daily oral administration of *β*-sitosterol for 7 days, the number of metastatic colonies in the lungs of B16BL6 melanoma cells was prominently less than that of the control group, which might be the result of enhanced immune surveillance activity evidenced by the increase of NK cells and immune response cytokines such as IL-12 and IL-18. Aloe-emodin is a natural anthraquinone derivative of many Chinese herbs, and it is reported that the administration of aloe-emodin dampened the burden of lung metastasis through the inhibitory effect on capabilities of invasion and migration of MDA-MB-231 cells [28, 29]. With a substitution of methoxy group at position 4, formononetin (C_16_H_12_O_4_) has a promising anticancer effect through influencing a variety of mechanisms, which pertains to a class of 7-hydroisoflavones [30]. Zhou et al. found that formononetin dramatically declined the development of lung metastases at 20 mg/kg/day by suppressing MMP-2 and MMP-9 through PI3K/AKT signaling pathways [31]. With the inhibition rate of lung metastasis reaching 82.2%, catechin exhibited extraordinary power in weakening the invasion of B16F-10 melanoma cells by inhibition of metalloproteinases [32]. Luteolin, 3′,4′,5,7-tetrahydroxyflavone is a common flavonoid that exists in many types of plants including fruits, vegetables, and medicinal herbs [33]. A previous study showed that luteolin markedly inhibited lung metastases of breast cancer by reversing epithelial-to-mesenchymal transition via downregulation of *β*-catenin expression [34]. The research conducted by Cook et al. revealed that relatively low levels (10 *μ*M) of luteolin remarkably inhibited VEGF secretion in MDA-MB-231 (4175) LM2 cells, thereby suppressing the occurrence of pulmonary metastasis [35].

In all, there were 47 intersection targets obtained between SC and secondary malignant neoplasm of lung and 8 of them were regarded as hub targets after utilizing MCODE plug-in unit of Cytoscape, namely RELA, JUN, MAPK1, MAPK14, STAT3, IL-4, ESR1, and TP53.

RELA (transcription factor p65), which is the p65 subunit of nuclear factor NF-kappa-B (NF-*κ*B), participates in cancer development and metastasis by forming chronic inflammation. Upon the phosphorylation of S276 amino acid residue mediated by TNF (tumor necrosis factor), RELA leads to the promoter-specific methylation and transcriptional repression of tumor metastasis suppressor gene BRMS1 (breast cancer metastasis suppressor 1) via direct recruitment of DNMT-1 (DNA (cytosine-5)-methyltransferase 1) to chromatin [36]. BRMS1 was found to reduce metastatic burden to lung by at least 75% (*P* < 0.05) and decrease the ability of MDA-MB-231 cells to adhere *in vivo* and *in vitro*, demonstrating that RELA can induce lung metastasis by suppressing BRMS1 [37]. So far, the dispensing granule of SC has been proven to lower the expression of phosphorylated-NF-*κ*B p65 protein [38].

MAPK1 (mitogen-activated protein kinase 1) and MAPK14 both belong to MAPK, which plays a vital role in the switch from extracellular signals to intracellular responses [39]. Gagliardi et al. [40] observed that MAPK1 knockdown of SUM149 TNBC cells appeared restrained anchorage-independent colony formation and mammosphere formation, which indicated compromised capacity of self-renewal, migration, and invasion. Besides, the lung metastatic burden of SCID-beige mice injected via the tail vein with SUM149 shMAPK1 cells was predominantly lower than that of control mice. As the direct targets of miR-326 and miR-532-5p, MAPK1 participates in lung metastasis of hepatocellular carcinoma (HCC) cells due to tumor-associated macrophage infiltration, and it has been proven positively correlated with the expression of circASAP1, which was identified by comparing the circRNA sequence between common HCC samples and those with high metastatic potential [41]. MAPK14, commonly referred to as p38*α* MAPK, was proven to strengthen the formation of tumor-platelet aggregates that interact with the lung endothelium to form pulmonary metastases [42]. The extract of SC was reported to inactivate the expression of p38*α* MAPK [43].

JUN (transcription factor AP-1), a downstream factor of ERK pathway, is involved in gene reprogramming in many tumorigenic processes [44]. The blockage of c-Jun activity palpably weakened the invasion of laryngeal and hypopharyngeal squamous cell (LHSC) via a decrease of MMP-13, which is featured by the high propensity for lung metastasis [45].

Upregulated in approximately 50% of all human tumors, STAT3 (signal transducer and activator of transcription 3) is a key signaling protein engaged by a multitude of growth factors and cytokines to elicit diverse biological outcomes including cellular growth, differentiation, and survival [46]. According to the latest research, the mechanisms of STAT3 promoting lung metastasis are summarized as the polarization of macrophages and neutrophils and the activation of JAK/STAT3 signaling pathway. M1 macrophages and N1 neutrophils present antitumor property, while M2 macrophages and N2 neutrophils boost the proliferation of cancer cells [47]. The inhibition of STAT3 reversed the polarization of macrophages from M1 to M2, thus reducing HCC cell viability, proliferation, invasion, migration, and the formation of lung metastases [48]. Similarly, STAT3 activated the secretion of LCN2(lipocalin 2) from N2 neutrophils, which induced mesenchymal-epithelial transition of tumor cells via ERK/KLF4 signaling pathway and thereby facilitating colonization and metastatic outgrowth in lung under the circumstance of nicotine [49]. The activated JAK2/STAT3 pathway transcriptionally inhibited the tumor suppressor miR-506-3p in colorectal cancer (CRC) cells, which in turn led to pulmonary metastases [50]. Besides, lung metastasis of orthotopic BT474-TtzmR xenografts was suppressed by the inhibition of JAK2/STAT3 signaling pathway [51]. However, there is no direct and powerful evidence for SC downregulating STAT3, by now which needs further experiments.

Similar to STAT3, IL-4 (interleukin-4) assists lung metastasis mainly through M2 polarization of macrophages [52], which results in activation of epidermal growth factor receptor signaling in malignant mammary epithelial cells [53]. ESR1 (estrogen receptor) is closely related to the metastasis of various orthotopic cancers including breast [3], lung [54], and prostate [55]. Based on a clinical study involving 54,147 breast cancer patients, lung metastasis was more prone to occur in ER-subtype cases accompanied by worse prognosis [56] and luteolin stemming from SC has been proven efficient in suppressing lung metastasis of ER-negative breast cancer [57]. TP53 (cellular tumor antigen p53) is a classical protein with an anticarcinogenic effect, the loss of which renders colorectal cancer cells more likely to transfer to the lung [58].

The GO.BP enrichment analysis spotlights the fundamental position of response to oxidative stress in SC treating lung metastasis of malignant tumor. Lung colonies of B16F10 cells increased apparently caused by oxidative stress, assessed by p22phox and SOD mRNA levels and the NRF2 protein level [59]. In addition, Cao et al. [60] claimed that hypoxia facilitated HCC cells withstood oxidative stress and eventually promoted pulmonary metastasis by regulating TXN (thioredoxin) on HIF-2*α* (hypoxia-inducible factor 2*α*). It has been revealed that pretreatment of luteolin reversed oxidative stress induced by H_2_O_2_ in a dose-dependent manner [61].

Among the top 15 results of KEGG pathway enrichment analysis, the AGE-RAGE signaling pathway and microRNAs in cancer are the most relevant to lung metastasis of malignant tumor.

Synthesized during chronic hyperglycemic conditions or aging, AGEs (advanced glycation end products) are the products of a nonenzymatic reaction between the free reducing sugars and proteins, lipids, or nucleic acids [62]. The combination of AGEs and their receptor RAGE activates diverse signal transduction pathways, enhancing the progression of cancer. The AGE-RAGE signaling pathway was reported to stimulate the migration of human melanoma cells, and the inhibition of which quenched the spontaneous pulmonary metastases [63]. Nevertheless, there is no basic experiment exploring whether SC and its components can reduce lung metastasis by targeting the AGE-RAGE signaling pathway.

MicroRNAs (miRNAs) are small noncoding regulatory RNAs with sizes of 17–25 nucleotides and can be transported to distant organs by cancer-secreted exosomes to regulate local microenvironment for engraftment and colonization of circulating tumor cells [64]. miR-25-3p from CRC cells promotes vascular leakiness via adjusting the expression of VEGFR2, ZO-1, occludin, and Claudin5, consequently enhancing CRC metastasis in the lungs of mice [65]. Some research elucidated that luteolin exerted an inhibitory effect on migration and invasion of CRC cells by modulating miR-384 expression [66], which might be a novel strategy to prevent lung metastasis.

According to the results above, we found that the majority of hub targets and biological process including RELA, MAPK1, MAPK14, STAT3, IL-4, and oxidative stress are directly associated with inflammation, which is a key feature of premetastatic niche [8]. As the core factor, inflammation is also reported to regulate JUN [67], TP53 [68], ESR1 [69], AGE-RAGE signaling pathway [70], and miRNAs [71] in various disease types, which might in turn accelerate lung metastasis of tumor. In all, this study predicts prospective targets and signaling pathways in treating lung metastasis by SC.

Last but not least, limitations are also clear that further experiments are necessary to verify the results, and the studies cited in this paper are mainly based on animal or cell experiments, indicating deficiency of clinical research as proof. In addition, data of SC and lung metastasis could be more comprehensive by retrieving more literature and databases.

## 5. Conclusions

Integrating network pharmacology with molecular docking, our research elaborated that SC could prevent or reduce lung metastasis of malignant tumor to some extent by its active components through targeting RELA, JUN, MAPK1, MAPK14, STAT3, IL-4, ESR1, and TP53. Oxidative stress, AGE-RAGE signaling pathway, and microRNAs in cancer also play important roles in lung metastasis of malignant tumor, which are likely to be reversed by SC, laying the foundation for further experiments. Moreover, inflammation is the core factor that integrates all these targets, biological process, and signaling pathways together, which indicates that SC prevents or reduces lung metastasis mainly by dispelling inflammation.

## Figures and Tables

**Table 1:** Active components of SC.

Mol ID	Molecule name	OB%	DL
MOL000296	Hederagenin	36.91	0.75
MOL000033	(3S,8S,9S,10R,13R,14S,17R)-10,13-dimethyl-17-[(2R,5S)-5-propan-2-yloctan-2-yl]-2,3,4,7,8,9,11,12,14,15,16,17-dodecahydro-1H-cyclopenta[a]phenanthren-3-ol	36.23	0.78
MOL000358	Beta-sitosterol	36.91	0.75
MOL000392	Formononetin	69.67	0.21
MOL000417	Calycosin	47.75	0.24
MOL000449	Stigmasterol	43.83	0.76
MOL000461	3,7-Dihydroxy-6-methoxy-dihydroflavonol	43.8	0.26
MOL000468	8-o-Methylreyusi	70.32	0.27
MOL000469	3-Hydroxystigmast-5-en-7-one	40.93	0.78
MOL000470	8-C-*α*-L-arabinosylluteolin	35.54	0.66
MOL000471	Aloe-emodin	83.38	0.24
MOL000483	(Z)-3-(4-hydroxy-3-methoxy-phenyl)-N-[2-(4-hydroxyphenyl) ethyl]acrylamide	118.35	0.26
MOL000490	Petunidin	30.05	0.31
MOL000491	Angelicin	37.5	0.66
MOL000492	Catechin	54.83	0.24
MOL000493	Campesterol	37.58	0.71
MOL000497	Licochalcone a	40.79	0.29
MOL000500	Vestitol	74.66	0.21
MOL000501	Consume close grain	68.12	0.27
MOL000502	Cajanin	68.8	0.27
MOL000503	Medicagol	57.49	0.6
MOL000506	Lupinidine	61.89	0.21
MOL000507	Psi-baptigenin	70.12	0.31
MOL000006	Luteolin	36.16	0.25

OB: oral bioavailability; DL: drug-likeness.

## Data Availability

The data in this study are available from the first author upon request.

## Supplementary Materials

Supplementary Materials Supplementary Table S1. Molecular docking of hub targets and active components.
