# Supplementary material for: Unveiling Potential Mechanisms of Spatholobi Caulis against Lung Metastasis of Malignant Tumor by Network Pharmacology and Molecular Docking
Source: Evid Based Complement Alternat Med. 2022 Mar 21;2022:1620539. doi: 10.1155/2022/1620539 (PMC8959948; doi:10.1155/2022/1620539)
Supplement: Supplementary Materials — Supplementary Table S1. Molecular docking of hub targets and active components. [file 1620539.f1.docx]

Supplementary Table S1. Molecular Docking of Hub Targets and Active Components

|  | RELA- Aloe emodin | JUN-  8-C-α-L-arabinosylluteolin | MAPK1- Calycosin | MAPK14- Catechin | STAT3- Medicago | IL-4- Vestitol | ESR1- Catechin | TP53- Hederagenin |
| --- | --- | --- | --- | --- | --- | --- | --- | --- |
| Affinity (kcal/mol) | -7.9 | -6.2 | -7.5 | -8.9 | -8.1 | -6.7 | -7.3 | -6.9 |
| Number of hydrogen bonds | 4 | 7 | 6 | 3 | 5 | 4 | 5 | 2 |
| Residues of hydrogen bonds | SER-45, PHE-119, L YS-122, GLY-171 | ASN-299, SER-292, ASN-291(2), LEU-294, GLU-293, GLN-290 | ARG-201(2), GLU-203, LYS-205, VAL-93(2) | ASP-168, MET-109(2) | HIS-457, GLU-455, THR-440, LYS-370, ASP-369 | ASN-97, ARG-53(2), ASP-87 | LYS-362, ASN-359, TYR-328, GLU-542 | ARG-174(2) |
| Number of hydrophobic interactions | 4 | 1 | 3 | 6 | 0 | 4 | 7 | 5 |
| Residues of hydrophobic interactions | PHE-119, LYS-122, PRO-129, ILE-128 | LEU-294 | LEU-92, PHE-53(2) | LEU-75, LYS-53, VAL-38, ALA-51, VAL-30, LEU-107 | - | TRP-91(2), ARG-88, LYS-84 | ALA-546, ASN-359, VAL-355, TYR-328, GLU-542, PRO-333, ASN-407 | THR-170, THR-211, PHE-212(2), VAL-172 |
| Number of π-stacking | 0 | 0 | 0 | 1 | 0 | 0 | 0 | 0 |
| Residues of π-stacking | - | - | - | PHE-169 | - | - | - | - |
| Number of π-Cation Interactions | 0 | 0 | 0 | 1 | 0 | 0 | 0 | 0 |
| Residues of π-Cation Interactions | - | - | - | LYS-53 | - | - | - | - |
